# Guy's Hospital Reports

**Published:** 1837-10

**Authors:** 


### Art. III.

*Guy's Hospital Reports.* No. IV. *April,* 1837. Edited by G. H. Barlow, M.A. and L.M., and J. P. Babington, M.A. &c.—*London,* 8vo. pp. 310; with numerous Plates.

The present Number of this work is perhaps the most valuable that has yet appeared; and proceeding, as it does, from the officers of one hospital only, offers a striking exemplification of what might be expected from the co-operation of the officers of all the London hospitals in carrying on a publication of this kind. Highly creditable as the volume now before us is to the zeal, industry, and scientific knowledge of the gentlemen connected with Guy's Hospital, the very excellence of the papers contained in it seems to us to afford a melancholy presage that the work cannot be long-lived; as it appears impossible that a sufficient supply of materials, worthy of the now-established reputation of the work, can be long expected from so limited a body of contributors. We shall, however, make the most of the good while it lasts; and, in the present article, propose to give as full an account of all the papers in the present Number as our limits can possibly allow. In doing so, we shall, as usual, exercise perfect freedom in stating our opinions, whether favorable or unfavorable to the respective writers. It is always much more agreeable to us to praise than to blame; but we owe a duty to our readers and to the profession, which obliges us to disregard our own feelings, whenever they can interfere with its performance.

The first paper is by Mr. Key, and is of great excellence and value. It is entitled "*A Practical View of Lithotrity; with Remarks on the Lateral Operation of Lithotomy.*"

Lithotrity justly ranks as one of the greatest improvements in modern surgery: there is no second opinion on this head. But its inventors have retarded rather than advanced its usefulness, by urging it forward as an operation designed to supersede, *in toto*, lithotomy. The over-zeal of its advocates has damaged its reputation more than the hostility of its opponents. The merits of lithotrity have been largely extolled; but its demerits have been, unfortunately, kept out of sight. The same, no doubt, may, to a certain extent, be said of lithotomy; and, between the biassed representations of the advocates of both, no accurate information was likely to be gathered respecting the merits or demerits of either, so long as the operations remained confined to individuals so opposed. It was only by both being put to the test by the same practitioner, that a fair estimate of their respective advantages and disadvantages could be arrived at. In this capacity of unprejudiced arbiter Mr. Key appears in the communication before us. Equally an adept in both operations, and having, consequently, no interest in the advocacy of one more than the other; being, moreover, a skilful and dexterous surgeon, and fully in the confidence of the public, his testimony is of much value. As foreseen by every disinterested mind, the time has arrived when there must be a drawn-battle between the advocates of lithotrity and lithotomy,—when both operations shall receive their due meed of praise,—and when each shall be practised according to its respective merits. The whole world admire and approve of lithotrity, and give the palm to its discoverers. They have however learned that it is not, as was at first represented, an infallible means of cure in all cases, and anxiously look forward to some conclusion regarding the extent of its applicability. Mr. Key's communication makes an important instalment towards the settlement of the question, and it is desirable that the example of candour which he has set should be followed by all possessing such opportunities. The solution of a great problem,—one involving the lives of thousands,—is at stake; and as, hitherto, nothing but the sunny side of each operation, and more especially that of lithotrity, has been exhibited to public view, we shall in future give more credit to him who owns to a few disastrous terminations of either, than to him who reports all in favour of one mode of practice.

Mr. Key gives to lithotrity every credit in its application to particular individuals, but he does not consider it, "as *indiscriminately applied* to all cases, either a less painful, more safe, or more successful operation than that of lithotomy;" and he contends "that, were lithotrity to be introduced to the entire exclusion of lithotomy, society would suffer by the exchange."

The knife will, leaving out the chance of accidents, cure all cases in which the lithotrite can be used, and it possesses the great superiority of being effectual in many instances where that instrument would be prejudicial. It cannot, therefore, be ever completely superseded.

In evidence of the failure of the lithotrite under particular circumstances, and of its success under others, Mr. Key reports some cases which he met in his own practice: other instances—drawbacks on the repute of lithotrity,—he knows of, but which, not being his own patients, he has abstained from reporting. Mr. Key's delicacy in the matter is, perhaps, laudable; but not so the secrecy of those who treated the diseases, and whose duty it was, for the benefit of the profession and of mankind, to have given them publicity.

The *first* case reported by Mr. Key was that of a gentleman on whom M. Civiale had operated. This gentleman, though reported as cured, died, in all the agony of stone in the bladder, in less than two months from the operation.

In the *second* case, a small stone was broken up by a well-known operator. This gentleman died of typhoid symptoms, shortly after his return to the country, from an abscess at the base of the prostate gland.

In the *third* case, after Mr. Key had failed in the operation of lithotrity, the stone was broken by a skilful lithotritist. In twelve months the patient died of diseased bladder; and, on examination, several stones were found.

The *fourth* case was a man of seventy, and declared by the lithotritist to be unfit for his mode of operation, on account of the large size of the stone, the age of the patient, the irritable condition of the bladder, and the enlarged state of the prostate gland. Lithotomy was practised; the patient was convalescent in a fortnight; and five years afterwards was well, and free from any symptoms of stone.

In the *fifth* case, the calculus was said to have been successfully broken down. The patient died not long after, and six or seven stones were found in the bladder, formed on the remnants of the original calculus.

The *sixth* case, a child nine years of age, was cut for the stone by Mr. Key. Six months after, another small stone was discovered. This was crushed, and the patient left town, *cured;* but died shortly after, in great suffering.

The *seventh* was a case of small stone, well adapted for lithotrity. The operation was performed at several sittings, and with success. The patient remained well two years after.

Case *eighth.* A gentleman of eighty, with irritable bladder and enlarged prostate. Objected to by a lithotritist. Cut by Mr. Key: twenty-seven calculi abstracted, and a cure accomplished.

Case *ninth.* A patient, after lithotrity, laboured under symptoms indicative of stone in the bladder. He was seized with bleedings from the bladder, and died in a few weeks. Several calculi were found, and each, when broken, was discovered to be formed on a fragment of crushed stone.

The *tenth* case is less satisfactory, as no dates are given. The gentleman was operated on several times by the lithotritist; and, at the time of Mr. Key's report, he appeared cured.

Case *eleventh.* Refused by the lithotritist, on account of the extreme irritability of the bladder and enlargement of the prostate gland. Lithotomy, nevertheless, was performed, and with success.

The *twelfth* case was that of a surgeon. The stone was crushed after three sittings, and the patient obtained entire relief from his disorder.

"Of these twelve patients, three were cured, and three underwent the operation of lithotomy. Of the remaining six who died, one sunk with abscess in the prostate gland, soon after the operation; four with protracted sufferings, in consequence of fragments remaining in the bladder; and one with disease of the bladder, brought on or aggravated by the operation." Mr. Key "believes that no surgeon conversant with lithotomy would expect so unsuccessful a result in these cases, if they had been all submitted to the knife. The three patients who could not undergo the operation of lithotrity, but were afterwards compelled to seek relief from the knife, recovered; and it is but reasonable to conclude that some of the unsuccessful cases would have had a better result, had they been subjected to the same operation." These patients were all treated by the most dexterous operators; all that lithotrity could effect was done for them; and the result clearly shows that lithotrity is not of universal application. We have ourselves known of instances in which incontinence of urine, irritable bladder, fresh stones, and even death in the course of a few months, have followed the employment of the lithotrite. But, as the patients were in other hands, and as no mention has been made of their misfortunes, we do not feel at liberty to speak of them further. We cannot however but feel, with Mr. Key, that, by keeping such cases in the background, the character of the operation of lithotrity suffers much disparagement. It is, no doubt, a most important invention, and if its advocates, instead of urging it forward to the exclusion of lithotomy, were to recommend and use it as an adjunct to that operation,—the only place which it is ever destined to hold,—they would much enhance the value of the improvement. A faithful narration of the varied results in all the cases which occur would give to each operation its proper station in usefulness; would enable us to determine, by comparison, where the one and where the other mode of practice should be adopted, and would save us from the well-merited imputation of perpetually floundering, through uncertainty, into error. Lithotritists should, themselves, endeavour to be the first to determine these points. Half the glory of the discovery will be lost by leaving to others the merit of pruning it down and fitting it in its proper sphere of usefulness.

Mr. Key, though a lithotritist as well as lithotomist, has, perhaps, taken rather a gloomy view of the new operation; but, as his only object has been to fill up a great blank in the history of lithotrity, left by the concealment of its disadvantages, he cannot be charged with partiality. We are infinitely more obliged to him for the narration of a few failures, with the explanation of the causes of these failures, than if he had reported one hundred unalloyed cases. By the term *cure*, Mr. Key and we understand a perfect freedom from the consequences of stone in the bladder,—not a temporary alleviation. The final result must be known before any case shall be deemed worthy of this epithet.

Since commencing the practice of lithotrity, Mr. Key has found that more than one-half the number of adults who have come under his care have been fit subjects for the operation; and that, in the majority of persons afflicted with calculus, it has decided advantages over lithotomy. One among the principal advantages which lithotrity has conferred upon surgery, is the early application which patients are induced to make for the relief of their disorder. The prospect of being cured without cutting is an important consideration with an invalid, and leads him to seek for an early investigation into his malady, with a view to its easy removal. Mr. Key has even found patients pleased with the discovery of a stone, under the impression that, by the new operation, they would be easily and certainly cured. The introduction of lithotrity has also led to an improved method of examining for the stone, by which calculi of a small size are more readily discovered now than formerly. Large stones are now very uncommon; and hence lithotrity will become even more employed as it is made more generally known.

Mr. Key reviews the dangers incidental to lithotomy, and endeavours to determine how far the new operation enables us to avoid them. There is no period of life at which lithotomy is impracticable; but infancy, the age for which lithotrity is peculiarly unfitted, is that in which the knife is most successful. Mr. Key considers "that it is difficult to mention any operation in surgery so uniformly successful as lithotomy is in children." He has cut a child for stone at the early age of sixteen months, and assisted at an operation when the patient had only completed its thirteenth month. The lithotrite can never become a substitute for the knife in children. Indeed, the latter is so safe that a substitute can scarcely be said to be required. The same rule holds good up to near the age of puberty. In the middle periods of life, the principal danger in lithotomy is hemorrhage; death from which, when it occurs, is usually gradual. Mr. Key is of opinion that loss of blood has a bad effect on the issue of all surgical operations, and he rarely ever bleeds a patient before any severe operation. In this practice the author is, we believe, joined by most surgeons of experience.

Infiltration of the cellular membrane with urine is another danger. It occurs more frequently in the adult than the young subject, and may be either the result of injury done to the deep perineal fascia, or of an unhealthy form of inflammation, which breaks down and destroys the barrier that nature opposes to the infiltration; or, more often still, of the violence committed in dragging the stone through the incision in the gland. Mr. Key considers that contusion of the prostate is worse than its laceration; and that, in this respect, there is a resemblance between the effects of lithotomy and lithotrity; for in both it is the violence done to the neck of the bladder that destroys the patient, in the majority of fatal cases. The author explains the circumstances under which such violence is usually produced, and gives some useful suggestions on the best mode of avoiding them.

Another source of danger, to which a wound in the perineum exposes the patient, is peritonitis; an affection which is generally independent of injury to the membrane, and would appear to be propagated to it by the cellular tissue of the perineum.

Lithotomy is almost free from the risk of leaving fragments in the bladder, but it exposes the rectum to danger. A wound is, however, seldom inflicted on this bowel by an expert hand. Mr. Key has only known of one case of the kind, and in that no untoward circumstances followed. Another aged individual is mentioned by him, in whom, in consequence of an abscess, the urine continually flowed from the bladder into the rectum, without being productive of any bad consequences.

These cases, although they may have induced Mr. Key to attach little importance to a wound of the rectum, are insufficient to authorize such as a general conclusion. We have ourselves known of a case in which an accidental wound of the rectum in lithotomy healed, without retarding the cure; but, on the other hand, we have heard of another where the very opposite result followed. We have seen a third, wherein the formation of a fistulous aperture between the bladder and rectum, by sloughing, was followed by distressing and fatal consequences; and a fourth, in which a fistula in perineo, the consequence of stricture, burrowing its way into the rectum, became the immediate cause of the patient's death. The circumstances on which all these varieties of results depend are not yet sufficiently made out.

"The after-consequences of the operation, of an untoward kind, may be summed up in the accidents of fistula in perineo, impotency, and incontinence of urine." Of these Mr. Key has had but little experience. Incontinence is the most common, and is more likely to occur in children than adults. In the former, as they approach the age of puberty, the power of retention becomes increased.

In reference to *lithotrity*, the author considers the circumstances that render it practicable, the mode of performing, and the dangers attending it. He also describes the several steps of the operation, and lays down rules to assist in determining which operation is the more eligible in each particular case.

The size of a calculus forms no objection to lithotrity, but, as several sittings will be necessary, it is essential that in such a case the bladder should be free from disease and not very irritable, as otherwise the fragments cause so much irritation as to render the repetition of the operation not only dangerous but impossible. We should pay more regard to the general condition of the patient and of his bladder than to the size of the stone. Adult age is that best adapted for lithotrity; as, here, the urethra is sufficiently large to admit instruments and to discharge the fragments without difficulty; the prostate is healthy and the neck of the bladder sound, and little exposed to the dangers of inflammation; the bladder itself is also in a vigorous state for the expulsion of the fragments. The aged subject, however, is not less adapted to the operation than the younger adults, if he be free from the common incidents of age. If the parts in the aged are sound, the operation is especially successful in them. The large size of the urethra allows fragments of extraordinary size to pass. Mr. Key possesses a piece of calculus discharged by an elderly gentleman, eight lines and a half in length and four and a half across.

"The state of the bladder is, perhaps, of all the circumstances that the lithotritist has to consider, the most important, and one on which the propriety of performing the operation will mainly hinge. Three conditions of this organ are necessary; and these must be ascertained by preliminary observations and trials, before the operation is determined on:—1st. It must be capable of holding a sufficient quantity of water to facilitate the working of the percussor. 2dly. It must be free from that extreme irritability that often attends the latter stages of calculous disorders; and 3dly, Not prone to inflammation from slight excitement." (P. 35.)

Amongst those who apply for advice soon after the symptoms have begun to declare themselves, it is rare to meet with any difficulty in injecting water sufficient for the purpose of giving space for the operation: not so, when the bladder becomes morbidly irritable from the continued presence of the stone. Mr. Key has remarked that the most formidable symptoms, such as copious discharge of dark-coloured, phosphatic mucus, great frequency and pain in making water, quick pulse, furred tongue, rigors, &c., will often yield to a system of diet and medicine so as to bring a patient, unexpectedly, into a condition to bear the operation of lithotrity. Regarding rigors after the use of instruments, the author makes an important distinction in them. Where the rigor is not followed by pyrexia, it indicates nothing more than the degree of irritation produced by the sound, and may not stand in the way of the operation; but, when followed by pyrexia, it is an evidence of local inflammation; and such a state is most unfavorable to lithotrity. The disposition to inflammation is often kept up by improper food, especially drink, and is indicated by a plethoric state of the system and flushed countenance. Such a condition is unlike that state of bladder or kidneys, the effect of commencing disorganization, and may be overcome: there is not the healthy aspect of mere temporary excitement; there is generally more pallor of countenance, amounting to a pasty state of the skin, with an occasional streak or patch of colour.

A morbid condition of the cervix vesicæ—a principal source of irritability of the bladder,—may be often diminished by the occasional and careful introduction of a large instrument; more especially, if assisted by a regulated diet, and alkaline and saline medicines. Without such preparation, a high and dangerous inflammation will, even in a person enjoying good health, most likely be kindled up by the employment of the lithotrite. Mr. Key reports an instructive case in which he operated without sufficiently preparing the patient. Hemorrhage and retention of urine followed. He opened the neck of the bladder by the lateral operation, and gave exit to six ounces of coagulum and a small calculus: the patient recovered perfectly. The gentleman suffered less pain from the knife than the lithotrite; but, as Mr. K. states, when persons are well prepared, the pain given by the lithotrite is inconsiderable.

All the author's practical directions respecting the operation are excellent, and deserve to be borne in mind. The bladder should not be too much distended. The end of the catheter should be fully introduced into the bladder before injection; as, otherwise, the eyes of the instrument may be obstructed by the sides of the prostate gland, especially if enlarged. The rectum should be emptied by an enema an hour before the operation, as a loaded state of that bowel may render the bladder irritable and indisposed to expand under injection. When obstruction arises from unusual fulness of the prostate, the operation should be deferred. Force ought to be particularly avoided. Before the blades of the instrument are expanded, the surgeon should feel assured of its being passed beyond the prostate, and fairly lodged in the bladder. Such precaution is particularly necessary where any enlargement of the prostate exists. In all cases the end of the lithotrite should move freely in the bladder before any attempt is made to open the blades. The best test of the crushing process being conducted properly is the absence of pain and hemorrhage. When the bladder is not irritable, the seizure of the fragments may be repeated from five to eight times at one sitting, and a large portion of the stone reduced to a state fit for expulsion. The sensations of the patient become the surgeon's best guide in repeating the operation at the first setting. Healthy persons are generally able to attend to their usual occupations after the proper use of the instruments. The pain caused by the stone will not unfrequently be relieved after one sitting, and the operator may be misled by the false estimate made by the patient of his feelings, leading him to defer, too long, the steps necessary for the complete removal of the fragments. While the bladder continues sound, these fragments may remain unchanged, but, when otherwise, they become nuclei for a rapid deposit of phosphates, and by their size give rise to much aggravation of distress.

Mr. Key has planned and practised an improvement designed to effect the desirable object of breaking up the stone at one sitting, and of thereby saving the inconvenience of the frequent application of the lithotrite. The invention consists in the addition of a net to be thrown round the stone, when caught by the blades, by means of two hidden branches. He considers that, by the net, the fragments left by the first act of crushing can be all readily brought in succession between the teeth of the instrument; and he reports four cases in which success followed its use. We will not, at present, venture farther, in criticism on this point, than to remark that the addition of the net appears to add considerably to the complexity of an already truly complicated piece of machinery; and no one knows better than Mr. Key that simplification of operations, rather than additions upon their difficulties, is a great desideratum in surgery. As we could not fully convey Mr. Key's meaning, in reference to his improvement in the short space allotted to us, we must refer those who may be desirous of trying it to the plates and description of his instrument, as given by himself. We cannot do better than to terminate this abstract of Mr. Key's instructive paper by a quotation from his concluding paragraph, which contains advice of the highest importance.

"In trying to lay down rules for the performance of an operation like lithotrity, that requires and depends upon manual skill, I feel that the best advice the young practitioner can receive is, to gain a familiar acquaintance with his instruments, by frequently operating on the subject. No rules can supply the place of practical dexterity; and this, as in all other mechanical arts, is to be acquired only by continued practice. Theory will do little for the lithotritist. If he expects that a general acquaintance with its principles, and with the action of an instrument, will render him expert in the performance of the operation, he will find that he will obtain experience after repeated failures, and at the expense of severe suffering and hazard to his patient." (P. 55.)

The second paper, which is, perhaps, one of the least important in the volume, contains "*Observations on the Diagnosis of Pneumonia*, by Dr. Addison," who very properly protests against our resting in satisfied inaction, even in the present very advanced stage of our knowledge of the diagnosis of thoracic diseases. "The main object of this brief communication (he says,) is to make some addition, however trifling, to the ordinary means of diagnosis, since experience has forced upon me the conviction that there are few acute diseases more frequently mistaken or overlooked than pneumonia, to the detriment of the patient and the no small embarrassment of the practitioner."

In his preliminary remarks, Dr. Addison notices a class of cases which he regards as sometimes constituting a species of phthisis, although in reality of inflammatory origin, and not tuberculous.

"In some instances, however, when the albuminous matter thrown out is of the more plastic and organizable kind, it fails to be entirely absorbed, and part of it permanently remains. Under these circumstances we find it, at an after-period, either in small, detached, and more or less rounded masses, or more extensively and more irregularly diffused through the pulmonary tissue. When distributed in small insulated portions, I believe it to constitute one of the forms of albuminous deposit, indiscriminately called tubercles; whereas, when more extensively and irregularly diffused, it has, in like manner, been regarded as a form of tubercular infiltration. The history, however, of the patient's case, in many instances, as well as the local appearances themselves, lead me to the conclusion that they are merely the result of a previous attack of pneumonia." (P. 60.)

Evidence of their inflammatory origin is found, he thinks, in the thickening and adhesions of the adjacent pleuræ, which sometimes, as well as the surrounding pulmonary tissue, present also a darkened appearance; and he regards this view of the origin of these albuminous deposits as serving to explain why they are much less uniformly found in the apices of the lungs than ordinary tubercles. He believes in their occasional conversion into calcareous masses, as well as in their still more frequent softening and breaking down into vomicæ, which, as the vital influence by which they are retained in their integrity is extremely slender, readily takes place when inflammation is casually set up around them; an event of frequent occurrence in weak and exhausted constitutions.

The characteristic symptoms of pneumonia enumerated by Laennec, independent of those cognizable by auscultation and percussion, are—obtuse and deep-seated pain in the chest, dyspnœa, hurried respiration, cough, and peculiar expectoration. But all these, it is well known, may be absent; and Dr. Addison has persuaded himself that all are really absent in the simplest form of the disease, and that its presence is hence very frequently altogether overlooked. The cases of pneumonia in which the above symptoms are met with, though confessedly the most frequent in practice, are, according to him, really cases of complication.

"In *simple pneumonia*, after chilliness, shivering, feebleness, and depression, the patient experiences, for the most part, strongly marked symptoms of febrile reaction, giddiness, confusion, and sometimes intense pain in the head; occasionally delirium, especially towards night; *the skin acquires a pungent heat*, generally accompanied by dryness, more rarely by moisture; the pulse is full and strong, perhaps labouring and sluggish; the face is usually more or less suffused with a livid flush, accompanied by an expression of distress; the tongue is foul; its substance is more injected than in ordinary phlegmasiæ, and in a short time it manifests a tendency to become dry and brownish; the respiration is somewhat hurried, but *there is seldom any very obvious cough or expectoration, and sometimes none at all;* in short, the whole assemblage of symptoms bears a most striking resemblance to those of a severe attack of common continued fever of the typhoid type, for which it is so repeatedly mistaken. If this form of the disease occur in moderately good constitutions, and is overlooked, especially if stimulants be administered on the supposition of its being a severe case of typhoid fever, it very commonly happens that the general prostration increases, the delirium or oppression of the brain is aggravated, the tongue gets dry and black, and the teeth covered with sordes; the breathing becomes more hurried, occasionally with a frequent slight hacking cough, and now and then a little bloody expectoration; the pulse gets flaccid, frequent, and feeble; and at length the patient dies." (P. 62.)

To an attentive observer, some difference even in the *general* symptoms of the two affections is perceivable. In pneumonia, the countenance, though congested and somewhat distressed, has not the dejection and stupidity so remarkable in fever, and the patient, on being roused, even though delirium should exist, commonly evinces a clearness and vigour of intellect not found in the latter affection; nor is the contrast between the vividly injected tongue and its white or grey fur so striking; or we have, as Dr. A. expresses it, more the tongue of a phlegmasia than of a fever. "But, of all the symptoms of pneumonia, (says Dr. A.) the most constant and conclusive, in a diagnostic point of view, is a *pungent heat* of the surface." By this symptom alone, the first stage of pneumonia may, he thinks, in most instances, be readily recognized.

With regard to this symptom, the bringing forward of which in a prominent point of view seems to have been the chief object of the present communication, we cannot help feeling that its value, even were we to admit its universal presence in pneumonia, (which our own experience does not authorize us to do,) is very seriously reduced (and especially just in the commencement of the disease, when such a diagnostic is most wanted,) by the circumstance of its frequency in very many other acute affections; amongst which Dr. A. himself enumerates continued fevers, and more especially those of children; the eruptive fevers, and particularly scarlatina; erysipelas, and some forms of renal dropsy; and the list might be much extended.

As to the cough and expectoration so commonly observed in pneumonia, Dr. A. strongly suspects that they depend, not on the inflammation of the air-cells themselves, but altogether on the accidental implication of the bronchial tubes; and that it is only when the smaller tubes are inflamed that we have the viscid expectoration, erroneously, as he thinks, looked upon as a characteristic of pneumonia in general. The complication, however, is admitted to be more frequently present than absent.

Pain, he conceives, is seldom felt in any position from pneumonia alone, in the absence of pleuritis and of inflammation of the bronchi; and when either of these exists, the pain which accompanies it is of a distinct and peculiar character, being acute and penetrating if the former be present, and consisting in a sensation of burning, tearing, and general soreness and rawness, if the latter.

The paper next in order is a very valuable one, by Mr. Alfred S. Taylor, lecturer on Medical Jurisprudence at the hospital, and author of the admirable elementary work on that branch of medical science. It is entitled "*Two Cases of Fatal Poisoning by Arsenious Acid, with Remarks on the Solubility of that Poison, in Water and other Menstrua.*" It contains many very important practical hints, and displays much talent for scientific investigation.

The discrepancies which prevail amongst authors, even of the highest authority, as to the degree of solubility of arsenic in water, both at ordinary temperatures and at the boiling point, are very remarkable. The researches of Mr. Taylor on this head, which is one of such obvious importance in a medico-legal point of view, seem to have been conducted with such a degree of accuracy and caution, as to entitle them to implicit confidence.

The first case is that of a female, of twenty-five years of age, who had swallowed about forty grains of arsenic in coarse powder, mixed with water. In about an hour afterwards she became very ill, and vomited, had pain in the stomach, great thirst, and a sense of constriction in the throat. She was brought into Guy's Hospital about seven hours after the poison had been taken; emetics and mucilaginous drinks having been administered, with a slight temporary alleviation of the symptoms, in the interim. Her countenance was now pale and anxious, the extremities cold; there was occasional vomiting, *great thirst;* tongue moist, but very red; *the pain in the stomach was not great, and not increased by pressure;* the pulse 134, very irregular, but rather full. There was no pain in the head, and her mental faculties were unimpaired. A mixture of equal parts of oil and lime-water was introduced into the stomach by means of the stomach-pump, with a view to envelope any residuary particles of the poison, as well as to sheath the coats of the organ. The stomach was first well washed out with this mixture, and then about three ounces of it, along with forty drops of tincture of opium, were injected into it, and allowed to remain.

She died about *fifteen* hours after having taken the poison, in a slight momentary convulsive fit, having become for some time previously very restless. *Extreme thirst* continued throughout to be the most prominent symptom, but was unaccompanied by any increase of pain in the stomach or any headach, and she was sensible almost to the very last.

On dissection, some particles of arsenic were found in the stomach, notwithstanding the care which had beeu used for its removal during life. The rugæ of this organ, which were very prominent, were vascular on their edges; whilst there were here and there dark-coloured patches of blood extravasated beneath the mucous membrane. There was great vascularity in the upper part of the stomach, and three distinct vermilion-coloured lines running between the pyloric and cardiac orifices.

"The most striking morbid changes, however, in the stomach existed near the larger curvature towards the pyloric extremity. Here there was a large prominent oval patch of thickened membrane, about three inches in length and two inches in breadth. This patch was in the first instance covered with a dense layer of opaque mucus, with difficulty separable, containing small quantities of arsenic diffused in a white pasty mass. When the surface was washed, it was seen to be of a yellowish colour in the centre; and it was surrounded by a dark margin, as of extravasated blood. At this part the coats of the stomach were at least three-quarters of an inch in thickness. There was no trace of ulceration or corrosion in any part of the mucous membrane. The peritoneal coat was slightly injected."

The small intestines contained a great quantity of viscid mucus tinged with bile, and presented obvious marks of inflammation, especially in the jejunum.

The period at which the symptoms commenced was, as Mr. T. remarks, about the ordinary one; viz. an hour after taking the poison. The amount of pain was unusually slight; for it is for the most part so excruciating as to be compared by the sufferers to a fire burning within the body, and is almost always aggravated by pressure. It is well, however, to know that this symptom may be occasionally almost entirely absent, a fact already alluded to by Orfila and Christison; and that death in such cases may be even more than usually rapid, and the cadaveric changes consequently less considerable. In such, likewise, cerebral symptoms are for the most part manifest. But here, again, this case was peculiar: the mental faculties remained unimpaired to the last, and there were neither comatose symptoms nor convulsions till just the very last moment.

*Thirst* has been observed by Mr. T. to be a very prominent symptom in three other cases that fell under his observation, as well as in this one, and he thinks it has scarcely been dwelt on enough by Christison and other British toxicologists. Professor Martini looks upon it, when conjoined with dryness and constriction of the fauces, as affording the most certain evidence of irritant poisoning.

The lime-water was not employed with any view to its chemical antidotal powers, which are now generally exploded; the arsenite of lime being easily redissolved in water, if any acid or even slight excess of the poison be present, and being in itself, though insoluble in pure water, capable of acting deleteriously.

The average period of death in cases of poisoning by arsenic, when it is taken in any considerable quantity, may be stated at from six to twenty-four hours. But it is well to know that there are cases on record where the fatal event has occurred within two hours and a half, or even, as would seem from a case given by Remer, within a still shorter period.

In Mr. T.'s case there was neither ulceration nor gangrene; and he suggests the probability of effusion of blood beneath the mucous membrane having been occasionally mistaken for this latter phenomenon. The thickening of the coats of the stomach he is disposed to consider as of much more frequent occurrence than their perforation.

In regard to the analysis for the detection of arsenic: when it has been taken in the form of a coarse powder, we may sometimes shorten our labour much by availing ourselves of its greater specific gravity, as compared with the viscid fluid in which it is entangled. Thus, by diluting the contents of the stomach largely with distilled water, so as to diminish their consistency, and agitating for a moment violently in a tall glass vessel tapering to a point, and then letting it stand, the heavier particles settle to the bottom, and may, without further trouble, be submitted to examination.

Into the details of the analysis we have not room to enter, but would refer our readers to the paper itself as a good example of the manner in which investigations of this kind should be conducted. The sesquisulphuret of arsenic having been formed, was identified by its insolubility in the mineral acids, by its perfect solubility in liquor ammoniæ, and finally by its yielding a ring of metallic arsenic when slowly heated with four times its weight of black flux. Mr. Marsh's apparatus for the detection of minute quantities of arsenious acid, as well as of the soluble salts of the metal, by passing hydrogen gas, in its nascent state, through the fluid containing them; burning the arseniuretted hydrogen so obtained, and holding a clean plate of glass over the flame, so as to have formed thereon a stain of metallic arsenic,—is spoken of with much approbation, as affording a very delicate and satisfactory test. The precautions requisite to ensure its accuracy are stated at length, and the objections which have been made to its use satisfactorily answered. "In respect to its delicacy, (says Mr. T.,) I have obtained sublimates from $\frac{1}{100}$ of a grain in 45,000 parts of water."

In the second case (for the details of which we have not space,) there was vomiting and violent diarrhœa, followed by a tendency to coma, with great restlessness and cramps in the legs; a rapid, feeble, and irregular pulse; pain in the abdomen, increased by pressure, &c. Death ensued within seventeen hours. The mucous membrane of the stomach was found inflamed throughout, and in many parts ulcerated, and especially on the summits of the rugæ. No arsenic was found within the organ, though about an ounce was supposed to have been swallowed; a circumstance which is attributed to the extent to which the vomiting and diarrhœa had been carried. The intestines, as well as the œsophagus, larynx, and trachea, were all highly inflamed. The treatment consisted in the use of the stomach-pump and of albumen, the exhibition of an emetic of sulphate of zinc, and copious draughts of tepid water to encourage vomiting.

The occurrence of ulceration within so short a period is unusual. Christison thought it was rarely to be met with, save where the case had lasted at least two days. Menstruation was present in the above instance. Cases are on record where this phenomenon has been recalled long before its stated period by arsenical poisoning, though there was no evidence that such was the fact here.

Some obstruction was thrown in the way of the analysis of the fluids rejected from the stomach during life, by the presence of sulphate of zinc. The operation with sulphuretted hydrogen failed; but Marsh's "hydrogen test," already alluded to, rendered the presence of arsenic quite obvious, though the quantity operated on was extremely minute.

The copper and the silver test are both rejected by Mr. Taylor, except when operating on a clear solution of the poison in which there is certainly no organic matter, nor any foreign salt, alkaline or metallic.

Common salt, which is so very likely to be met with in the stomach, mixed with arsenic, not only destroys the action of the silver test, but renders that of the copper also ambiguous. Now, though, by a complicated process, this substance may, it is true, be got rid of, yet the "hydrogen test," as well as the reduction of the precipitated sulphuret, are so entirely satisfactory as to render further and more operose proofs altogether superfluous. The operator must not, however, forget that the presence of a metallic salt, such as the sulphate of copper or of zinc, or tartarized antimony, so often exhibited as emetics in these cases, might give rise to some ambiguity in the reaction of sulphuretted hydrogen. So likewise, if a compound poison were swallowed, (such as a mixture of corrosive sublimate with arsenic,) the analysis might be seriously embarrassed, unless the operator were aware of the effects of sulphuretted hydrogen on particular metallic salts. But such mixtures do not at all interfere with the results afforded by Mr. Marsh's apparatus.

The paper concludes with some remarks on the solubility of arsenious acid in water and other menstrua. The affinity between arsenic and water at low temperatures is very slight. Even when boiling water is poured on it, and *allowed to cool*, the quantity dissolved, though greater than in the preceding instance, is still remarkably small, and much less than the quantity retained in a cold saturated solution prepared by *boiling* the arsenic and water together *for several hours*. Various explanations of this have been attempted, none of which are satisfactory. Thus

Fischer, a German chemist, conceived that the arsenious acid underwent a change during the process of boiling, one portion of it being rendered more soluble by abstracting oxygen from the remainder; and, in conformity with this view, he asserts that the portion which separates by crystallization on cooling, is very much less soluble than common arsenious acid. But Mr. Taylor shows satisfactorily that this explanation is erroneous; the diminution of solubility in the crystallized portion being at most very inconsiderable, and only such as may be ascribed, as well as the change of colour to a dusky brown or yellowish colour, to the altered arrangement and increased force of cohesion of the crystalline particles. There is, in fact, no conversion of arsenious into arsenic acid. "We know (says Mr. T.,) that the effect of heat in rendering solids more soluble in water is due to this agent increasing the force of affinity between the solid and the liquid with some bodies; and arsenious acid is a direct instance of this. It is necessary that the heat should be for some time applied, in order that the affinity of water for this body should be raised to its maximum degree."

Guibourt, one of the chief authorities on the subject of the solubility of arsenious acid, and who is quoted largely by Berzelius and Dumas, noticed a great difference in solubility between the opaque kind (common arsenic) and the transparent, and in favour of the former; a result which is, however, as we have seen, quite at variance with Mr. Taylor's experience. Guibourt's results with regard to the solubility of the opaque variety are as follows:

| | | | | |
|---|---|---|---|---|
| 1000 parts of water, | at 60°, | dissolve | 12.5, | or $\frac{1}{80}$. |
| 1000 — | at 212°, | — | 114.7, | or $\frac{1}{8}$. |
| 1000 — | at 212° (cooled to 60°) | | 29.0, | or $\frac{1}{34}$. |

An extraordinary difference exists amongst those chemists who have experimented on this subject. Thus, 1000 parts of temperate water dissolved, of arsenious acid, according to Despretz, $\frac{1}{20}$;—according to La Grange, $\frac{1}{24}$;—Bucholz, $\frac{1}{50}$;—Guibourt, $\frac{1}{80}$;—Hahnemann, $\frac{1}{96}$;—Spulmann, $\frac{1}{96}$;—Ure, $\frac{1}{333}$;—Klaproth, $\frac{1}{400}$;—Fischer, $\frac{1}{1200}$.

This singular discrepancy is attempted to be accounted for by Mr. Taylor, on the supposition that some of these experimentalists made their deductions from actual solution in cold water, and others from solutions formed by boiling, and allowed to cool before examination.

The difference in regard to the solubility in water at the boiling point, as stated by different experimentalists, is also startling. Thus, 1000 parts of boiling water dissolve, according to Guibourt, $\frac{1}{8}$ of their weight;—Bucholz, $\frac{1}{12}$;—Klaproth, $\frac{1}{13}$;—Ure, $\frac{1}{14}$;—La Grange, $\frac{1}{15}$;—De la Métherie, $\frac{1}{24}$;—Vogel, $\frac{1}{60}$;—Beaumé, $\frac{1}{64}$;—Navier, $\frac{1}{80}$;—Nasse, $\frac{1}{200}$.

This disagreement, Mr. Taylor thinks, may be explained by supposing that a heat of 212° may have been applied for different periods of time, and that the specimens of arsenious acid employed varied in their degree of solubility. The specific gravity of a mass of arsenious acid which had been kept for four years, and was perfectly opaque, but still slightly crystalline in its newly fractured surface, was found to be 3.529; whilst that of a recently prepared specimen, which was perfectly transparent, but had a slightly yellowish tinge, was 3.798.

Arsenious acid is soluble in water, alcohol, and oils. Mr. T.'s experiments were directed to the two former menstrua. We must here again limit ourselves to his general deductions.

"From the whole of these experiments, I may perhaps be permitted to draw the following conclusions:

"1. That hot water, allowed to cool from 212° on this poison dissolves less than $\frac{1}{400}$ of its weight, or about $1\frac{1}{4}$ grains to each ounce of water.

"2. That water boiled for an hour in this substance dissolves $\frac{1}{24}$ of its weight, or rather more than twenty grains to each ounce.

"3. That this water, on perfect cooling, does not retain more than about $\frac{1}{40}$ of its weight, or twelve grains to the ounce.

"4. That water boiled on arsenious acid to the most perfect state of saturation, after having stood six months, holds dissolved about $\frac{1}{38}$ of its weight, or thirteen grains to the ounce.

"5. That there is no observable difference in the solubility of the transparent and opaque varieties of arsenious acid.

"6. That water at ordinary temperatures will dissolve from about $\frac{1}{1000}$ to $\frac{1}{500}$ of its weight, or from half a grain to one grain to each ounce of solvent, according to circumstances.

"7. That the presence of organic matter in a liquid is an obstacle to the solution of the poison. Thus, hot tea and cold porter will not take up more than about half a grain to the ounce; while hot coffee and cold brandy do not dissolve more than a grain to the ounce. (Alcohol dissolves more than twice that quantity, or, according to Brande, eight times.)

"There are two points of practical importance which these experiments suggest in relation to the analysis of organic liquids suspected to contain arsenic, especially when the liquids have been obtained from the stomach or intestines: first, to dilute the liquid considerably with water; and, secondly, to boil the liquid thus diluted for at least two or three hours. By attending to the first point, we in a great degree destroy the effect of organic matter in impairing the solubility of the poison; and by the second we insure the solution of every portion of poison which may be present." (P. 102-3.)

It was observed, in the experiments alluded to, that the arsenic which was in the form of a fine powder occasionally floated in part on the surface of the cold as well as the boiling water, in a film or in small lumps; and in part adhered to the sides of the vessel, whilst the remainder sunk to the bottom. The rapidity of boiling made a considerable difference in the quantity dissolved; water which boils violently taking up as much in half an hour as that kept gently boiling in twice that time. The length of time during which the boiling is kept up also makes, all other things being equal, a notable difference in the quantity dissolved.

It is deduced, from Mr. T.'s experiments, that very nearly the same quantity of arsenious acid is taken up by hot water allowed to cool, and by cold water poured on this substance in powder, provided the vessel containing the cold water be frequently agitated. It is a curious, and hitherto an unexplained fact, that water should retain so much more of this poison, as from ten to twenty times the quantity, when *perfectly cooled* from a *boiling* saturated solution, that it will take up at common temperatures without heat.

When administered in the state of a perfectly transparent saturated solution, as in a case which was the subject of trial at Mayence, about two years ago, its effects may prove most rapid, and at the same time no trace of arsenic be found, on analyzing the contents of the viscera after death.

As to the menstrua ordinarily employed, suicides generally prefer water, whilst murderers select some liquid article of food, such as tea, coffee, brandy, or gruel, which they conceive calculated to conceal the supposed disagreeable taste of the poison. It has not, however, if pure, any of that acrid, caustic flavour attributed to it in several medico-legal works, but (as stated by Christison,) merely a faintly sweetish taste. The solubility of arsenic is much impaired by the presence of organic matter. No certain opinion can be expressed respecting the solvent powers of gruel, broth, or liquids of a similarly viscid nature: "they mechanically suspend the particles of arsenic exactly in proportion to their viscidity, and thus a very powerful dose of poison may be administered in a small quantity of liquid." Hahnemann found that water impregnated with mucus or milk dissolved the poison with difficulty.

In taking our leave of this excellent paper, we would once more strongly recommend its careful perusal to all persons interested in the examination of the medico-legal questions connected with the subject of arsenic; as several of the points likely to be raised in the course of the trial of cases of alleged poisoning by this mineral are here discussed, and settled on the only true basis—that of cautious induction from well-conceived and carefully executed experiments.

Then follows a paper by Mr. T. W. King, "*On the Safety-Valve Function of the right Ventricle of the Human Heart; and on the Gradations of this Function in the Circulation of Warm-blooded Animals.*"

The object of this essay is to shew that the auriculo-ventricular valve at the right side of the heart, in man and some other classes of animals, is so constructed, as, upon certain occasions, to perform imperfectly the office of closing the passage between the ventricle and auricle, during systole. On these occasions the tricuspid valve is supposed to act as a *safety-valve*, the explanation of which, in the author's words, is as follows:

"The veins, being more or less influenced by their own number and capacity, by the position of the body, by cold, compression, repletion, and respiration, the blood is brought to or collected in the right ventricle in varying quantities; and on occasions of the most copious influx, the cavity becomes distended; upon which, portions of the tricuspid valve are drawn aside, an aperture of reflux is produced, and the force of the ventricle is diverted from the pulmonary circulation at the moment when the lungs might otherwise be overwhelmed."

In the preceding quotation we have enumerated various powers which propel the blood through the veins to the right side of the heart, and cause fulness of the right ventricle; "but the most persistent and important power of the venous circulation that we have to consider (says Mr. King,) consists in a pulsatile wave derived from or through the capillary system. When the body is at rest in the horizontal position, what are the means by which the veins transmit their contents, from all extreme parts, to the centre of the circulation? To this enquiry I should not hesitate to reply: that the chief force is the ventricular impulse, propagated, through the arterial tubes and the capillaries, into the veins of every part."

Mr. King doubts that any propelling power resides in the capillary vessels; "yet would not absolutely deny to these vessels an operation similar to that of the heart, namely, a passive diastole under the influx from the arteries, and an active subsequent systole, together with an apparatus tantamount to valves, by which the onward current of the circulation is sustained. . . . In many vascular parts, common size injection passes readily from the veins into the arteries; which is a fact much opposed to the idea of a valvular action in the capillaries. We cannot positively deny to the capillaries a propulsive peristaltic or vermicular action, but yet it seems almost inadmissible." On this subject we may remark that the circulation in many of the simpler animals seems to require a propelling power in the capillaries: for instance, in fishes, from whose single ventricle the bronchial arteries arise, terminating in the bronchial veins, which coalesce to form the aorta. This vessel again becomes continuous, by its capillaries, with the veins of the body, which return the blood to the heart. It can hardly be supposed that the ventricular impulse is continued throughout this double series of vessels, each so minutely divided. The same observation may be applied to the portal circulation of the liver in man, and other animals in which the portal veins pass through that organ. The well known fact, also, that soon after death the arteries are found generally empty, and the veins full, requires some other explanation than is supplied by the propelling power of the left ventricle.

Mr. King believes in the existence of a venous as well as an arterial pulse. By means of a "sphygmometer," consisting of a fine capillary lever with a long index radius, made by drawing out a piece of sealing-wax into a thread about two inches long, and, with a little tallow, fixing this across a vein, on the back of the hand, so that nine-tenths of its length might project on one side of the vein, Mr. King states that the pulsation of the veins becomes very manifest, especially under circumstances of repletion. "The action of this little sphygmometer," he says, "has been witnessed by many; and I scarcely think that any exception can be fairly made to its application, to prove the existence of a pulse or undulating current in the veins. Applied to an artery, it plays freely and quickly: on a vein its movement is very steady, and only visible when neatly adapted; whilst, if applied to any other part, no motion is produced." We have tried this experiment, but with different results from those described by Mr. King. We have not been able to perceive any motion indicating a venous pulse.

The circumstances illustrating accumulation in the right ventricle are stated as follows:

"That the right ventricle readily becomes turgid from obstructions to the pulmonary circulation is well seen in experiments upon the living heart, *in situ.* The right side begins to swell with the first disturbance of the respiration, whether the lungs are kept distended . . . or suffered to collapse; . . . while the reduced state of the left side of the heart clearly shews that the lungs admit but little blood." The accumulation of blood in the right side of the heart and its distention, in the act of dying, the frequent hypertrophy and morbid dilatation of the right ventricle, are adduced as proving that this "cavity is subject to a certain internal expanding force, at least, in a state of disease: a force that cannot be supposed slight, when it is observed to operate effectually against the natural contractile power, and often against a very considerable increase of substance and power in the muscular sac. The dilated and hypertrophic right ventricle is continually found in death fully distended with blood; and I am inclined to think that this dilated ventricle is diminished in some degree after death, by the tonic contraction common to all muscles. It seems reasonable to infer, that in the natural states of the circulation, some degree of distention is of much more common occurrence."

The circumstance which led the author to the study of the present subject was a post-mortem examination, in which the right ventricle was found morbidly dilated, and which afforded "conclusive evidence that the right ventricle is liable to dilatation, and that the dilatation deranges its valves. The last proposition is thus explained. The cavity is formed by the solid septum of the heart for its inner wall; and by a thinner, more extensive, and yielding layer of muscle for its outer or right wall; whilst each of these walls affords points of attachment to the cords of the valves. The distention of the cavity, operating chiefly upon the weaker paries, carries it outwards, together with the cords and curtains attached to it; and the parts of the valve being drawn from the proper plane of their valvular adjustment, the backward communication into the auricle remains open."

The author's description of the tricuspid valve in the human heart, abbreviated, is as follows. The valve consists of three parts, or curtains: the fixed curtain occupies the left margin of the aperture in apposition with the solid wall, from which arise all the cords affixed to this portion of the valve. A second, or anterior, curtain is attached at the anterior and right edge of the opening, having one free border forwards, and another backwards in the ventricle. The cords of this portion of the valve are inserted, some into the solid, and others into the yielding wall of the ventricle, near its centre; where also is attached a muscular band stretching across the cavity between the two walls, and called by Mr. King "the *moderator band* of distention." The third or right *curtain* is situated on the right side of the aperture posteriorly: and the greater part of its cords are attached, by the intervention of muscular columns, to the yielding wall. "Having concluded," Mr. King says, "that all parts of the valve in connexion with the yielding wall of the ventricle are affected by dilatation, I venture to call them *curtains*, *cords*, and *columns* of *distention*. The *anterior* and *right curtains*, then, are parts of distention, together with the cords and fleshy columns attached to the *yielding wall*."

The experiments of the author, in illustration of his views, consist in injecting the ventricles through their respective arterial trunks; a portion of the auricles having been previously removed, in order that the closing of the valves might be seen. He found that, generally, the mitral valve prevented the reflux of the fluid employed, while the tricuspid, closing less perfectly, allowed some to pass. As regarded the right side of the heart, much depended upon the state of the organ. If flaccid and distended by fluid, the valve closed imperfectly: if, on the contrary, the muscles were firm, and over-distention was prevented, the tricuspid valve was found to perform its office well.

There are, we think, two objections to these experiments; first, that the flaccid and easily distended right ventricle of the dead heart is a most inadequate representative of the same part in the living subject: secondly, that a very small pipe was used for injection, "rather less than the fourth of an inch in diameter, and often still smaller;" whereas, in order to imitate the systole of the living heart, a pipe ought to have been employed equal in diameter to the trunk of the artery, and care should have been taken to imitate the contraction of the ventricle by compressing it gradually with the hand, during the process of injecting. By employing these precautions, we have found the tricuspid valve to perform its office remarkably well, allowance being made for the flaccid state of the papillary muscles, (called by Mr. King "*columns of distention*,") in the dead heart.

There can be no doubt, however, that if the right ventricle be very much distended, either in the dead or in the living body, its valves will be inadequate to close the passage to the auricle, until the distention has been partially got rid of, and that, previously to this, in the living heart, a portion of the blood will be driven back into the auricle.

The second and third parts of this paper are devoted to the examination of the tricuspid valve in mammalia and birds; but we are unable to follow the author into the details of this portion of his essay. He arranges the individuals of each class into series, according to the degree of valvular perfection in the right ventricle; and endeavours, but we think not satisfactorily, to shew in each an adaptation of the anatomical structure to the habits of the creature.

The author's style is, in many respects, faulty; much too prolix, and sometimes obscure; and the *ninety-one* notes, which are to be found in the *seventy-five* pages, of which his paper consists, prove to us either a want of natural connexion in the subjects, or deficient powers of arrangement in the author. We would observe, also, that the terms "columns, cords, curtains, parts of distention," strongly savour of that rage for extravagant nomenclature, by which certain anatomical writers of the day are mainly characterized.

This article is followed by "*An Experimental Inquiry respecting the Process of Reparation after Simple Fractures of Bones*," by Mr. Bransby Cooper.

This gentleman, at the suggestion of Sir Astley Cooper, has undertaken a series of experiments to determine, with accuracy, the phenomena which occur in the reparation of fractured bones. We cannot, until the inquiry is completed, do more than state the plan adopted by Mr. Cooper, and express our hope that the result will prove instructive. A great deal has been already done on the subject; but it must be admitted that all parts of the process of reparation are not yet sufficiently understood.

The thigh-bones of rabbits were those selected by Mr. Cooper for experiment; and the bones were fractured in such a manner as to inflict the least possible injury in the soft parts. The animals were killed at different successive periods, and the dissection was performed in each about one hour after death. A description of the appearances is given, illustrated by well-executed drawings, which afford an instructive view of the several changes which occur. The conclusions which Mr. Cooper, from his enquiries, arrives at are nearly as follows.

The first effect of the fracture is extravasation of blood between the cancelli, and into the surrounding parts: by this, when the blood coagulates, the limb is stiffened, and farther bleeding prevented. There is little farther change for the first twenty-four hours. On the third day, the limb is rendered still more rigid by the effusion of lymph, the product of inflammation, between the bones and amongst the surrounding muscles. From this period the effusion, which is at first gelatinous, gradually thickens, forming a distinct tumour around the fracture, limiting motion, and preventing the muscles being irritated by irregular ends of the bone. After this, the extravasated blood is absorbed; the last remains of it being that interposed between the fractured ends of the bone. Mr. Key is of opinion that this coagulum becomes organized, and assists in the reparation.

About the sixth day, the effused matter has the appearance, firmness, and elasticity of cartilage, and by its contraction tends to bring the hitherto separated ends of the bones, even though overlapping, parallel and in contact with each other. At the broken part, the periosteum is absorbed; but elsewhere it is thickened, and more loosely connected with the bone than with the textures around. The denuded part of the bone becomes softened and more vascular. The first deposit of bony matter takes place on the surface of the old bone; and from this Mr. Cooper infers, that the osseous system alone is in such cases the source of the bony deposit. He supposes that "the surrounding textures so far assist in the reparation of a fractured bone as to induce approximation, limit motion, and diminish the irritability and contraction of muscle; whilst the osseous system itself deposits the earthy matter essential to hardness, the grand characteristic of bone." Mr. Cooper finds an argument in favour of this view in the fact that bones little organized, such as the cranium or neck of the femur, are badly able to work out their own reparation. From Mr. Cooper's experiments, it appears that, in rabbits, earthy matter is deposited in the cartilage so early as the ninth and sometimes the seventh day. Sections of such cartilage, placed on glass and dried, exhibit at this period the white osseous particles in the form of minute hard specks.

The only two points of novelty which Mr. Cooper's experiments would appear to suggest are,—the power of the callus to replace the deranged bones, and the secretion of the new osseous matter by the vessels of the original bone, exclusively. With respect to the first suggestion, it appears to us that there are other circumstances in connexion with the period of reparation in cases such as those experimented on by Mr. Cooper, which should, as well as the contraction of the callus, be taken into consideration, in accounting for the power of self-adjustment of the fragments; and, at all events, it would be well to have the matter cleared up, lest it be prematurely used as an argument in favour of slovenly treatment, or of the practice of leaving broken limbs without any treatment whatever, to heal the best way they can. As to the second point, it may, as being a mere physiological question, safely lie over for farther consideration. We may observe, however, in passing, that the fact of the secretion of bony matter commencing in the textures immediately next adjoining the original bone, is not now for the first time announced; and that some farther proofs than those adduced by Mr. C. are necessary before we can go the full length with him in believing that the whole mass of new osseous formation is derived exclusively from this source. The farther experiments and observations promised by Mr. Cooper, will, no doubt, set these important matters in a right point of view.

It would have given us sincere pleasure if we could have bestowed on the "*Reports of Obstetric Cases, with Observations*, by Dr. Ashwell," more commendation than we fear it will be in our power to do. We cannot compliment the author greatly either on his style or on the general character of the observations; although it is true that the paper contains practical remarks of value, and some curious cases. It is but justice to the author to state, that many parts of this report are obviously not the composition of Dr. Ashwell himself, but of those gentlemen who, as pupils or clinical clerks, had the management of some of the cases.

We are surprised to find it stated that in "*Inflammatio Oris Cervicisque Uteri,* cupping on the loins, anodyne injections, mild laxatives, with hyoscyamus, and absolute rest in the recumbent posture, comprised the whole of the treatment." Was there no trial of mercury, or of leeches applied directly to the part, or the nitrate of silver, or the warm bath?

The cases of "*irritable uterus*" were treated with "the hip-bath, hyoscyamus and camphor, assisted by the employment of anodyne injections and suppositories, . . . while tonics, as the calumba, cascarilla, and quinine, were administered, to maintain the powers of the constitution." In these cases, also, we think the remedies above mentioned might have been useful, as also guiacum and iron, which latter especially has frequently, in our hands, appeared to be the agent to which the patient was ultimately indebted for her complete cure. It would, moreover, have been satisfactory to have been told how many patients affected with these two complaints were cured by the treatment adopted.

All the cases of Leucorrhœa were treated with astringents and tonics: but no examination is reported to have been made of the state of the uterus and other parts concerned: are we to conclude that the treatment was adopted in reference to the mere discharge, without any thing being known of the condition of the parts from which it proceeded?

In Menorrhagia the ergot of rye is stated to have been of great service, and was employed, both internally and *externally,* "in the form of injections," (p. 206.) We do not understand what is meant by the latter expression.

We have in some of our preceding numbers had occasion to notice more than once the subject of Polypus of the Uterus, and have consequently anticipated the discussion of some points in which we differ from Dr. Ashwell. There are however some of Dr. A.'s views of this disease, both pathological and practical, which seem to call for further notice in this place. He details four cases of polypus successfully operated on by the ligature. Dr. A. excludes altogether the use of the knife in such cases; but we cannot admit, as the readers of the articles referred to are aware, that he entertains this preference "in common with English practitioners." In common with many, unquestionably, and some of these of great name, his preference is shared. Still, as formerly stated, several practitioners in this country decidedly prefer the use of the knife in many cases of polypus; and we think this preference particularly justified where the tumour is of the hard fibrous kind, the pedicle small, or of moderate size, or where the polypus protrudes, or nearly so, from the vagina, so that its stalk can be brought into view. In these cases, we consider that the knife is infinitely to be preferred; and as to the occurrence of so much hæmorrhage as might require the plugging of the vagina, surely the inconvenience of that simple and effectual proceeding is productive of much less annoyance and danger to the patient than the repeated tightenings of the ligature, the foul putrid discharges, the presence of the instrument in the vagina for so many days, and the risk of dangerous irritative fever.

We would say decidedly that a much greater number of accidents have, on the whole, followed the use of the ligature than that of the knife; the latter may be, and often is, difficult to use, and there is the risk of hæmorrhage; but where a ligature is applied and a week or ten days elapse before the pedicle is cut through, it is by no means the safe operation that is generally supposed: that it does most frequently happily succeed, we know by repeated experience, but we have also seen the contrary too often, to allow us to join in the exclusive commendation of this mode of proceeding.

It is asserted by many as a reason for using the ligature, that its application *always* puts an end to the hæmorrhages and is not productive of any pain. Now, neither of these statements can be admitted as universally true; the hæmorrhages are not always arrested by the application of the ligature, although in the great majority of instances they are so; and there is one kind of polypus which always gives pain when its pedicle is constricted, namely, that variety in which the proper tissue of the uterus is continued into the stalk of the tumour, or, as it sometimes is, into its body.* In a case tied by Dr. M'Farlane at Glasgow, on the application of the ligature the patient "complained immediately of acute pain, which in a few minutes became so severe that she could hardly be persuaded to submit to its continuance," and subsequent tightenings of the ligature reproduced the pain.†

In case 4, p. 238, Dr. Ashwell judiciously availed himself and with success of the powers of the ergot to cause the expulsion of the polypus from the cavity of the uterus, and to bring it within reach of his ligature; we think he might have done the same with advantage in the preceding case, instead of attempting to tie the polypus within the uterine cavity, by which he incurred an unpleasant failure, and caused the patient a hæmorrhage which reduced her to the brink of the grave. It may be mentioned here, that occasionally the administration of ergot in such cases has not only effected the descent of the polypus, but has caused its strangulation and removal. This we ourselves have witnessed, and Dr. M'Farlane has recorded a very interesting case‡ in which the same fortunate result followed the use of this remedy.

Dr. Ashwell's remarks (p. 239,) on the structure of polypi, shew, in our opinion, a deficient acquaintance with the nicer details of the subject, and of the investigations of others both here and on the continent. He maintains that the hæmorrhage comes from the polypus and not from the uterus: 1st, because, in his own cases, it ceased immediately on the tightening of the ligature; 2dly, because Mr. Langstaff related a case where a patient lost her life from the repeated bleedings of an undiscovered uterine polypus; 3dly, because a polypus examined by Dr. A. and Mr.

* See Cruveilhier. Anat. Pathol. liv. xi. pl. 6, fig. 1, 2, 3.

† Vide an excellent article on this subject by Dr. M'Farlane in Glasgow Med. Journ., vol. I. p. 424.

‡ Ibid. p. 414.

Sibson was proved to have vessels in it: and he moreover considers these facts as tending to prove that an uterine polypus cannot be the same as a fibrous tumour of the uterus.

Now, surely Dr. Ashwell knows that a fibrous tumour in the substance of the uterus is constantly productive of profuse and repeated hæmorrhages, and yet, as he himself says, "it is rare to meet with a hard tumour that bleeds," for this cogent reason, that the hard or fibrous tumour of the uterus has no vessels in it to yield blood.

We should think that Dr. A. must have seen polypi removed whose surface was smooth and perfectly unbroken throughout, and which had yet by their presence caused the system to be fearfully drained of blood; and he must be aware that the hard white polypus has not a single blood-vessel in its structure. Twice recently we have seen the substance of polypi of this description divided by the hook tearing through it, in the attempt to draw them down, and not a single drop of blood flowed from them; nor could a trace of a vessel be discovered in either of them, by the most careful inspection after their removal. Yet, in both these instances, the patients had been the subjects of profuse hæmorrhages.

Dr. A. wishes to deny the identity of uterine polypi with the hard fibrous tumours of the uterus, which, he says, has lately been maintained by some eminent pathologists. Between these, however, he is "disposed to believe that there are, occasionally, points of similarity, especially between large, old, and condensed polypi and these tumours; but it is erroneous to view this similarity as at all complete, or universally existing."

We are not aware that any eminent pathologists have maintained, as a general rule, the identity of uterine polypus and the fibrous tumour of the uterus: such a view cannot be entertained for a moment; but Dr. Ashwell's opinion, that they are always dissimilar, is equally untenable. The white membranous lines he considers as peculiarly distinctive of the fibrous tumour of the uterus, and as not to be found in the polypus. But Cruveilhier (liv. 24, pl. i.) proves to us that a polypus may possess these as perfectly as an uterine tumour; and we think there is hardly a large museum in which the same fact may not be verified: we ourselves have seen it repeatedly; and, in the two instances above noticed, of polypi without blood-vessels, the structure was completely that of the uterine fibrous tumour.

Among the peculiarities noticed by Dr. Ashwell as contradistinguishing the two diseases, he mentions the possession of *sensibility* by the hard tumour, and the want of it in the polypus; and the *vascularity* of the latter and non-vascularity of the former. But we have already shown that a polypus may be as destitute of vessels as the other variety of tumour. We are surprised to find Dr. A. asserting that "the hard tumour most frequently grows externally, *seldom* encroaching on the cavity of the uterus." We thought its doing so a matter of such common observation as to be familiar to every one; and so it has been spoken of by Cruveilhier, Dupuytren, and many others. But what need of dwelling on particular points, when we have good reason to believe, with the distinguished authorities just named, that the fibrous tumour of the uterus not unfrequently becomes the uterine polypus, simply by descent and the consequent formation of a stalk. "There are," says Cruveilhier in his remarks on pl. i. liv. xxiv., "hard polypi, which consist of an hypertrophy of the tissue of the uterus: there are others which consist of fibrous tumours developed underneath the mucous membrane of the uterus or close to it, which grow towards the uterine cavity, sometimes remaining enclosed in that cavity, and at other times passing through the os uteri and descending into the vagina." And Dupuytren uses almost the same words when speaking of the fibrous tumours of the uterus. "These tumours (he says,) are often situated at the internal surface of the uterus, and are either merely projecting into the uterine cavity, or are completely pediculated, and these are the most common, constituting the real fibro-cellular polypi, &c."*

Of the six cases of polypi reported by Dr. Ashwell, three were in women who had had children, one in a woman long married but never pregnant, and two in single women: it is not stated whether these latter had cohabited with the other sex or not. This proportion, though on a very small scale, is satisfactory as shewing the fallacy of the commonly entertained notion that a much greater proportion of old maids are attacked with these complaints. On this subject Dupuytren has given us a table on a much more extended scale, which we subjoin.

Of 58 women affected with polypus
- 54 were married, or being single had cohabited.
- 4 only were unmarried and supposed not to have had sexual intercourse.

—
58

Of 51 women, there were

| | | |
|---|---|---|
| 39 | married, and with from 1 to 10 children, | } 42. |
| 3 | unmarried, but with children, | |
| 8 | married, but without any family, | } 9. |
| 1 | unmarried, who had cohabited but had not had any child . . . | |
| — | | |
| 51 | | |

The proportion of instrumental cases given by Dr. A. is small, and therefore bespeaks laudable practice: in 627 deliveries the forceps was used twice and the vectis twice; that is, for both, once in $156\frac{3}{4}$: the crotchet only once in the 627. Altogether instruments were resorted to only five times, or once in $125\frac{2}{5}$. The mortality of the children born under presentations of the nates or feet is enormous, being no less than nine out of eleven. For this, however, it would be quite unfair to impute blame to Dr. A. as these cases were principally under the care of pupils. Of the 627 women delivered, six are reported to have died, or one in $104\frac{1}{2}$. This appears to us fortunate practice, considering the class of patients attended.

Of 22 cases of carcinoma uteri there occurred between the age of

| | | |
|---|---|---|
| 30 and 40 | — 8 | cases. |
| 40 " 50 | — 6 | " |
| 50 " 60 | — 6 | " |
| 60 " 70 | — 2 | " |
| | 22 | |

* Leçons Orales, tom. iii. p. 243.

All these women we are informed were married, and, except two, had been mothers; seven were of light complexion and fifteen dark.

Eleven cases of ovarian disease and one of ascites are detailed, which do not afford much ground for comment. The author's remarks on this group of cases are judicious, and coincide with the results of our own experience.

"The statistics of these cases are instructive. In nine out of the twelve patients, there was deranged menstruation. Eight were or had been married; four were single. Two had not been mothers; and the remaining six had produced only twenty-two children; fifteen of the number having been borne by two women; facts not altogether unimportant, where the ovaries are structurally diseased. The progress of ovarian dropsy is extremely uncertain, and the effect of treatment is not less so. Occasionally, the disease advances by almost imperceptible degrees, and, for years, is scarcely at all regarded: suddenly, however, and without any appreciable cause, the malady not unfrequently displays great morbid activity, and paracentesis is performed, to obtain transient and slight relief. All remedies, excepting the extirpation of the diseased viscus, participate in this general inefficiency; and we cannot but regret that the curative means are so few and so feeble. Negative treatment, or, in other words, an attention to the general health, avoiding, as much as possible, constitutional excitement and ovarian irritation, promise most favorably for the patient. The cases adduced, and many others, sufficiently attest the powerlessness of medicine; and as to the radical cure, it is too truly hazardous, to be more than very rarely even thought of. It is true, that many patients pass through a tolerably long and comfortable life with a large ovarian dropsy; and more might enjoy this immunity from suffering, if marriage and parturition were avoided, and if they could be induced rigidly to practise self-denial and abstinence." (P. 226.)

A case of hard tumour of the uterus is related, which acquired a great size and weight (26lbs.), and in which iodine appears to have been of service. In the efficacy of this powerful remedy Dr. A. expresses, and we think justly, his unabated confidence in the treatment of uterine and ovarian tumours.

At page 242 a case is reported by Mr. Joseph Ridge, in which, for the cure of a "purulent discharge from the lining membrane of the uterus," in a girl of nineteen, that organ was twice injected with warm water by Dr. Ashwell: by this, violent and of course dangerous inflammation was induced; but the original disease disappeared with the cure of the factitious inflammation. We must, however, be permitted to doubt both the accuracy of the diagnosis and the propriety of the practice in this case: and it is but doing justice to Dr. A. to state that in regard to the latter point he is himself doubtful. The remarks on this and several other cases do credit to his candour and impartiality.

We have next, an interesting case of adhesion between the walls of the vagina, causing retention of the catamenia, in which peritonitis and death followed a second operation for the division of the adherent surfaces. Dissection shewed that the accumulation had distended the uterine cavity; a fact worth noticing, because some persons doubt that such an effect is produced by retained menses: although we ourselves have seen the uterus acquire, under such circumstances, a size equal to that of the sixth month of pregnancy.

A case of "malignant disease of the external genitals complicated with pregnancy" is also reported by Mr. Ridge. After the completion of the seventh month Dr. Ashwell induced premature labour by puncturing the membranes; to which proceeding, under the circumstances, we do not object, but against the way in which the case was subsequently managed, we must strongly protest.

"In nineteen hours afterwards, labour-pains commenced; and during this interval her local sufferings had been much relieved, and she had enjoyed several hours' sleep. Every advantage was given, by restraining the rapid advancement of the fœtal head, for a gradual dilatation of the external parts; but as labour progressed, the labia became everted, and some dark grumous blood was discharged from the left. As the head was urged towards the outlet, it became evident that the latter would not allow its exit, without tearing away a considerable portion of the diseased structure, and giving rise to such a hæmorrhage as the enfeebled state of the patient's powers would ill sustain. At this time, Mr. Lever came to my assistance; and finding the head unusually firm and large, and that no pulsation was perceptible in the fontanelles, he determined to perforate the cranium. The greater portion of the brain escaped, with much blood; and the uterine efforts quickly expelled the collapsed head, the shoulders and nates gently following it. A slight laceration of the fourchette occurred, notwithstanding the firm support afforded to the perinæum, but it did not extend to the softer, or, rather, less scirrhous parts. The placenta soon followed. The uterus firmly contracted, and, excepting a slight oozing from the morbid growth, scarcely any blood was lost. The child was well formed, and judged to be a little beyond the seventh month." (P. 249.)

It would appear from this, that lest the woman, whose condition was already hopeless, might possibly sustain some injury, or lose some blood, and because "no pulsation was perceptible in the fontanelles," a living child, arrived at the period of viability, was destroyed. It will not, we presume, be pretended that the absence of pulsation at the fontanelles is any proof whatever of the extinction of fœtal life. It is but justice to Dr. A. to state that he does not appear to have had any hand in this proceeding; at least, he is not mentioned as having been present, but he notices it without censure in the *Remarks* appended to the case.

The next case, also one of placental presentation, reported by Mr. Jackson, seems to us a striking example of injudicious practice. The patient seems to have been left from the 18th December till the 22d January without any examination being made to ascertain her real condition, although she had, in the interval, profuse hæmorrhages and grinding pains. This was more especially the case on the 14th January, when dilute sulphuric acid with infusion of roses, opium, and the application of cloths dipped in cold vinegar and water to the lower part of the abdomen, were had recourse to.

"This was attended with success, as far as regarded the bleeding; though the pains continued at intervals till Friday night, the 22d, when suddenly there was another discharge of blood: her spirits became depressed; her pulse quick, and small; severe pains occurring every twenty minutes, accompanied with the expulsion of clots of blood: the liquor amnii was also trickling away. Availing myself of a pain, I examined, and found a small portion of placenta projecting over the posterior edge of the os uteri, which was yielding. I now sent for Mr. Lever. After his arrival, there was no further uterine effort, or bleeding; the pulse was 120, small, and the patient excessively low: on examination, he found the presentation, as stated—the os dilatable, and the head within reach. He ordered tinct. opii m. xxv., and enjoined quiet. During the two following days, the liquor amnii continued to escape: she was more comfortable; took her medicine; and, an anodyne being exhibited at night, she slept tolerably well. On Monday morning, although no subsequent hæmorrhage had occurred, there was sudden dyspnœa, with jactitation of the upper extremities; pulse quick, and small; no uterine effort; and every indication for a speedy emptying of the uterus. I ordered brandy, slightly diluted with water, to be administered to her, by means of a tea-spoon, every five minutes; and went for Mr. Lever, who immediately delivered her of a still-born child, by turning; Dr. Ashwell being present." (P. 257.)

It seems unaccountable that when Mr. Lever was called in on Friday night, nothing was done except giving twenty-five drops of tincture of opium, and enjoining rest; and that the woman was left to struggle until Monday morning without even the precaution of plugging the vagina. Then when she is in articulo mortis, and not till then, it is discovered that there is "every indication for a speedy emptying of the uterus," from which a still-born child is extracted. Transfusion was had recourse to, but ineffectually. Dr. Ashwell does not appear to have been informed of what was going on till it was too late, but, in his remarks he tells us that "this case is instructive as shewing that in cases of exhaustion there is something wanted to revive and re-establish the living principle, which the supply of blood cannot furnish." We think that the case is very instructive in another point of view, as shewing, that inert trifling, and procrastination in cases of such imminent danger, as are those of placental presentation, can lead only to the most disastrous results.

The last case contained in the report is one of pregnancy with imperforate uterus, drawn up by Mr. Tweedie. The details are carefully given, and under the circumstances we believe the incision made into the cervix was justifiable; though we think it not impossible that had a free venesection been premised and some further time given, an os uteri might have been found. Dr. Dewees mentions a case in which displacement of the os uteri by obliquity was mistaken for its absence; delivery was effected by dividing the cervix, as in Dr. A.'s case; and the woman was afterwards delivered of several children per vias naturales.* Dr. Ashwell is mistaken in supposing that the description of such an occurrence is original with him: Nägele has described a cause of dystocia in agglutination of the external orifice of the os uteri arising between the time of conception and labour;† and complete occlusion of the os uteri has also been recently met with, demanding the free use of the bistoury before the labour could advance,—the patient being twenty-three years of age, and the complete closure of the os uteri ascertained by ocular inspection as well as by the touch.‡

The only communication of Dr. Hodgkin in this Number is the "*Description of a remarkable Specimen of Urinary Calculus; to which is added, some Remarks on the Structure and Form of Urinary Calculi.*"

The specimens (two in number,) described by Dr. Hodgkin were taken from the bladder of a boy, two years of age. They were about the size of a pigeon's egg, of different shapes and of a whitish colour. The surface was soft, as if covered with a fleshy layer, which prevented the detection of the calculus by the sound during life. The texture composing the surface was not unlike that of some blighted, acephalocyst membranes. On making a section, it was discovered that the calculus was made up of several alternating layers; one set soft, like that on the out-

* Compend. Midwifery, 5th Edit. p. 126.
† Arch. Gen. de Med., Oct. 1835.
‡ Entbindung bei Vollkommen verwachsenen Muttermunde. Siebold's Journal für Geburtshulfe, 1835.

side; the others of an opaque, white substance, having a fragile earthy texture; but, although the earthy layers were so brittle as to be crushed by the act of making the section, the fragments were so effectually retained in their relative situations by the tenacity of the membranous layers, that the two portions into which the calculus was divided were able to retain their form and cohesion.

From an examination of this calculus, Dr. H. is disposed to offer an explanation of the nature of a specimen in Guy's Hospital Museum, not hitherto understood; the characters and history of which are somewhat of the same kind as that described. He supposes that the two specimens mutually explain each other. The patient, the subject of the Guy's calculus, was a lad who had been in the habit of passing a milky fluid, slightly tinged with blood, and taking, by coagulation, the form of the vessel in which it stood. Dr. Hodgkin is inclined to believe that the patient who produced the specimen of calculus described by him, as above, must at times, in consequence of the derangement of the kidneys or bladder, have produced urine having somewhat the character which had been constant in the lad who furnished the specimen alluded to; and that, by an alternation of such coagula with the deposition of phosphates, the peculiarity in question was produced. The explanation is a most rational one; and it would have been satisfactory if Dr. Hodgkin could have verified, by observation, the resemblance of the condition of the urine in the two cases, the supposititious existence of which has led him to such conclusion.

Dr. H. compares the irregularities and fractures observable in some of the layers of such calculi to the interruptions sometimes seen in the sections of a stratified country; irregularities which he considers to have been produced by the successive and alternate deposits of hard and soft materials, the result of differences in the condition of the urine at different times.

In following up this principle, Dr. Hodgkin offers some interesting remarks respecting the varieties observable in the form and composition of calculi in general, and gives a plate illustrative of his views, representing fourteen different varieties of calculi.

The last paper in the collection, though by no means the least considerable, is one by Dr. Bright, which well supports his long-established reputation for sound pathological views and talent for observing the phenomena of disease, and graphically detailing them. It is entitled "*Cases and Observations illustrative of the Diagnosis where Tumours are situated at the Basis of the Brain; or where other Parts of the Brain and Spinal Cord suffer Lesion from Disease.*"

Of the nine cases given, the first two are perhaps the most interesting. There is a strong resemblance between them, and they mutually throw light on each other. In both, a tumour existed just beneath the tentorium, attached to the petrous portion of the temporal bone, and pressing aside the pons Varolii. The subject of the first case was a middle-aged officer, who, in the autumn of 1829, when on foreign service, began to suffer from a periodic pain in the situation of the left superorbitary notch, recurring daily about dinner-time, and ceasing as regularly before he had half-finished his meal. At this time he met with a very severe accident, the nature of which is not stated further than that he was taken up, after it, stunned and senseless. Though immediately bled, his recollection appeared for a day or two after very much impaired, and he never completely regained his former state of health. He made frequent complaints of pains in the head, and of some weakness in the right leg. A few weeks afterwards he had an attack of bilious vomiting, after which the pain in the head and over the eye became worse and more frequent, and was accompanied by an occasional loss of sight, coming over him like a cloud, and lasting a few minutes. Shortly after this, the intermittent pain over the left eye was completely removed, in a single day, by taking two or three doses of sulphate of quinine, in rapid succession, two or three hours before the usual period of attack; and it never returned:—a new and convincing proof, if any were wanting, of the possibility of intermitting symptoms depending on a local disorganization, and that they are occasionally amenable, even when they have such a source, to antiperiodic medicines.

About half a year afterwards, the patient had another attack of bilious vomiting, soon after which he discovered one day that he had completely lost the sight of the left eye; that of the right also became very imperfect, whilst the weakness of the right leg increased, and the left began to lose power. He had lost the hearing of the left ear, many years before, by the shock of a gun in firing; that of the right was tolerably perfect. He complained much of pain darting through his head, which was relieved by cupping, blistering, and tartar-emetic ointment.

It was in November, 1831, that Dr. B. saw this gentleman for the first time. He appeared perfectly unconscious of surrounding objects; obviously rather from the impairment of the external senses, than from any notable defect of mental power; for "when, after much trouble, by writing words on his hand and by calling in his ear, and by other means, he was led to comprehend, he answered distinctly and without hesitation, but in the high-raised and ill-modulated voice which is usually observed in deaf people." He was able to stand; but, when he attempted to walk, even with support, it was with a short, feeble, tottering step. He had no incontinence of urine, nor had he as yet passed his fæces involuntarily. His sleep was tranquil, and he was not more drowsy than might be expected in a person deprived of his sight and hearing. His appetite was good. He had experienced, about a week before, a fit, in which he had become insensible for a time, and his countenance suffused, but without convulsion. "There was no room to doubt that organic mischief was established within the skull: all that was recommended was medicine to regulate the action of the bowels, and a few grains of the subcarbonate of ammonia, with compound infusion of gentian; his diet to be very plain, and no wine."

When Dr. B. saw him again, about three weeks after, a slight amendment seemed to have taken place; as he could see a little better, and walk, with support, three-quarters of a mile. He sometimes spoke of "a peculiar sensation in his head, attended with a sound as if grease had been thrown into the fire, making a whizzing noise, and then dying away, whilst at the same time a flash of light passed over his eyes." The use of the arsenical solution was suggested, with a succession of blisters behind the ears and to the nape of the neck; and a seton was shortly afterwards introduced in this latter situation. His symptoms, however, soon began to grow gradually worse, and he sank in about a year after Dr. B.'s first visit, who had rarely seen him in the interim; the paralysis and drowsiness having slowly augmented, and the mental faculties become much weakened. The sense of taste seems to have been entirely lost, "so that for some months he had not seemed to prefer one article of food to another, and he supped the most nauseous medicines with the same apparent unconcern as he did wine or any pleasant beverage. Towards the conclusion, the urine and fæces were passed unconsciously; there were frequent and profuse perspirations, and extensive sloughing over the sacrum.

On dissection, the glandulæ pacchioni were found very much enlarged, and penetrating to the external table of the skull; the arachnoid in their neighbourhood being opaque, and adherent to the dura mater. The ventricles were distended by a serous fluid, of which about four ounces were collected. The septum lucidum was much thicker and firmer than usual, and several small vesicles adhered to the choroid plexus.

"In attempting to remove the brain from the basis of the skull, it was found that the anterior portion of the cerebellum on the left side degenerated into a tumour, and adhered so firmly that it could not be detached without a scalpel, or employing considerable force, from the petrous portion of the temporal bone. The structure of this tumour was chiefly hard and unyielding, but in some parts softer; and the nervus trigeminus, or fifth nerve, was seen passing over it flattened and broad: nor did the tumour simply adhere, but the bone had become carious and pervaded by it, so that a softened cavity occupied a large portion of the petrous ridge extending towards the sella turcica."

The second case presents several points of coincidence with that just detailed, both in the symptoms and in respect to the fungoid growth found attached to the petrous portion of the temporal bone, appearing to grow from its cancellated structure, and pushing aside the tuber annulare and the left hemisphere of the cerebellum, compressing the medulla oblongata and pushing the fifth nerve upwards. " In both the disease has been marked by its gradual progress; has first showed itself by affections of the senses, and then slowly produced paralysis of motion or sensation in various parts, affecting the intellect little till an advanced period of the disease, and probably not till it had led to extensive serous effusion into the ventricle." In both the left ear lost its sensibility, not much less than twenty years before death; in one, from the concussion of a cannon; in the other, after a severe wound of the face from the bursting of a gun, by which the cheek-bone was much injured, and the temporal bone had probably suffered from the concussion.

The organic mischief in the neighbourhood of the left ear sufficiently accounts for the deafness on this side, whilst that of the opposite may be ascribed to the pressure communicated through the pons Varolii. The impairment of taste (a symptom which is the more interesting from the circumstance of its being rarely mentioned in such cases,) is ascribed to pressure made by the tumour on the fifth pair of nerves. The impaired functions of the senses, and especially of sight, preceded by a considerable time any remarkable loss of power in the voluntary muscles, or any diminution of the common sensibility. Something analogous to fits of congestive apoplexy occurred in both cases, as the irritation and embarrassment of the brain gradually proceeded. The serous effusion, and consequent changes in the ventricles, had doubtless a considerable share in impairing the muscular power, and inducing the feebleness and oppression of the intellect observed in the later stages.

Several cases are recorded illustrative of the effects of lesions in different portions of the spinal marrow. One of a female is alluded to, who had paralysis of the glosso-pharyngeal and laryngeal nerves, (and of them alone,) so that she could with great difficulty swallow, and was quite speechless; and the case of a man is also cursorily mentioned, who had evident disease in the superior cervical vertebræ, in consequence of which he lost the power of articulating except in a whisper, while at the same time the lower extremities were paralysed. Though he recovered in a great degree the use of his limbs under the use of setons, he left the hospital with the same defect of voice still persisting.—Two cases of paralysis, connected with disease of the atlas and dentata, follow. In such instances, if, as often happens, pressure be made by the processus dentatus upon the upper part of the anterior spinal column, "the power of voluntary motion is destroyed throughout the whole trunk and limbs; while the nerves on which the particular senses depend,—those from which the motions of the muscles of the face, the tongue, the larynx, and the neighbouring parts, are derived,—and the nerves of sensation throughout the body all remain uninjured, and consequently the respective functions of the parts unimpaired."—The great object of treatment in these cases is, by perfect rest and counter-irritation, to favour the formation of anchylosis. A very good case (Samuel Elom's,) is given, in which, in the course of eighteen months, this happy result seemed to have been almost attained, when the patient, by an act of imprudence, revived the morbid process, and speedily fell a sacrifice to it.

Rheumatism would appear to be a very frequent cause of these affections.

Another case is reported, in which there was diminished sensibility and power of motion in the lower extremities, and yet no pain on pressure could for a long time after be discovered in any portion of the spine, nor any irregularity in the spinous processes; and, as a slight vertigo co-existed along with exfoliation of a portion of the frontal bone, the nature of the lesion was long doubtful. Sloughs occurred on the nates and hips; and though at first they healed rapidly, on diminishing the pressure upon them by means of the water-bed, yet, as the general health became subsequently still farther deranged, they recurred, and the patient sunk under their irritation.

In the case marked No. 7, along with hemiplegia of the right side, there was difficulty, not only of articulation, but also of connecting words justly with their corresponding ideas; and, on death, extensive disorganization of the right corpus striatum was the principal lesion found; which was in accordance with Dr. B.'s previous belief, namely, that derangements of the articulation are connected with lesions of this part of the brain. In case 8 likewise there existed a similar difficulty in connecting words correctly with the respective ideas which they properly represent. The replies to questions were consequently in many instances very ludicrous. This patient recovered. The 9th and last case is that of a gentleman, of fifty-eight years of age and gouty habit, who fell to the ground in a fit of vertigo, and experienced a numbness of the whole of his left side for several weeks after. There was a very peculiar morbid sensation in the fingers, so that every thing felt as if it were gelatinous or unctuous: when he touched his bedclothes, they felt as if arrow root had been spilt on them. This delusive feeling came on about two months after the commencement of his illness. "It lasted for a short period, not many hours, and was succeeded by several optical delusions: he fancied he saw persons in the room, and that, as he walked the floor, he had to step over gates, and stiles, and railings, and his mode of stepping corresponded with this conviction; at the same time he was capable of transacting business, so that the illusion appeared most probably to arise from some morbid impression on the optic nerve, and not from a mental process." Occasional impairment of hearing and sight were amongst his symptoms. Having been cut off some months afterwards by chronic peritonitis, a cell, about half an inch long, was detected in the right optic thalamus, and the surrounding portion was found to be in an unnaturally soft condition.

These cases we regard as of the greatest importance in relation to pathology, physiology, and phrenology. Every additional communication of Dr. Bright confirms our previous opinions, that medical science, largely indebted as it is already to him, is destined to receive still greater benefits at his hands. He conducts his enquiries in the true spirit of the inductive philosophy.

---

